# Effects of added folic acid on growth performance, short chain fatty acid concentrations, and serum homocysteine concentrations in nursery pigs

**DOI:** 10.1093/tas/txag017

**Published:** 2026-02-16

**Authors:** Larissa L Becker, Julian Arroyave, Jordan T Gebhardt, Mike D Tokach, Jason C Woodworth, Robert D Goodband, Joel M DeRouchey

**Affiliations:** Department of Animal Sciences and Industry, College of Agriculture, Kansas State University, Manhattan, KS, 66506-0201, United States; Department of Animal Sciences and Industry, College of Agriculture, Kansas State University, Manhattan, KS, 66506-0201, United States; Department of Diagnostic Medicine/Pathobiology, College of Veterinary Medicine, Kansas State University, Manhattan, KS, 66506-0201, United States; Department of Animal Sciences and Industry, College of Agriculture, Kansas State University, Manhattan, KS, 66506-0201, United States; Department of Animal Sciences and Industry, College of Agriculture, Kansas State University, Manhattan, KS, 66506-0201, United States; Department of Animal Sciences and Industry, College of Agriculture, Kansas State University, Manhattan, KS, 66506-0201, United States; Department of Animal Sciences and Industry, College of Agriculture, Kansas State University, Manhattan, KS, 66506-0201, United States

**Keywords:** folic acid, growth, homocysteine, nursery pig, Zn

## Abstract

Recent research reported an increase in growth performance when feeding supra-nutritional (up to 18 mg/kg) levels of folic acid to weanling pigs. Therefore, two experiments were conducted to validate these responses in typical U.S. nursery pig diets. In Exp. 1, 360 barrows (5.5 ± 0.03 kg) were used in a 38-d study to evaluate the effects of added folic acid (Rovimix Folic Acid, dsm-firmenich, Plainsboro, NJ) with or without pharmacological levels of Zn on growth performance and fecal dry matter (DM). Treatments were arranged in a 3 × 2 factorial with main effects of folic acid (0, 20, or 40 mg/kg) and Zn (3000 mg/kg of Zn in phase 1 and 2000 mg/kg in phase 2 or no Zn other than 110 mg/kg from the trace mineral premix). Diets were corn-soybean meal-based and fed in 2 phases (phase 1 from d 0 to 9 and phase 2 from d 9 to 24). Overall (d 0 to 38), increasing folic acid decreased (quadratic, *P ≤* 0.003), average daily gain (ADG), and average daily feed intake (ADFI) with pigs fed 20 mg/kg having the lowest performance. From d 0 to 24, pigs fed diets with pharmacological levels of Zn had increased (*P ≤* 0.030) ADG, and ADFI compared to pigs fed 110 mg/kg added Zn; however, no differences were observed in overall performance. Fecal DM was greater (*P = *0.007) on d 24 compared to d 9. A trend was observed where pigs fed diets with added Zn had increased (*P = *0.080) fecal DM averaged across d 9 and 24. Fecal total short chain fatty acid concentrations were greater (*P = *0.053) on d 24 compared to d 9. Isobutyrate increased (linear, *P = *0.030) as folic acid increased; however, most samples had nondetectable levels. In Exp. 2, 350 barrows (initially 6.0 ± 0.06 kg) were fed corn-soybean meal-based diets, fed in 3 phases (phase 1 from d 0 to 10, phase 2 from d 10 to 23 and phase 3 from d 23 to 38), and consisted of increasing folic acid: 0, 5, 10, 20, or 40 mg/kg. Overall, ADG, ADFI, and G: F were reduced (quadratic, *P ≤* 0.049) as folic acid increased with the poorest performance observed when pigs were fed 20 mg/kg of folic acid. On d 10 and 23, blood samples were collected from 70 pigs to determine serum homocysteine concentration Homocysteine concentrations increased (linear, *P = *0.037) with increasing folic acid on d 10; however, no differences were observed on d 23 (folic acid × d interaction, *P = *0.069). In conclusion, added folic acid reduced growth performance with pigs fed 20 mg/kg having the poorest performance, and increased serum homocysteine concentrations.

## Teaser

Folic acid is necessary for one-carbon transfer metabolic reactions, including nucleic acid synthesis, amino acid metabolism, and remethylation of homocysteine to methionine. Increasing added folic acid decreased growth performance and increased serum homocysteine concentrations in weanling pigs.

## Introduction

Folic acid (vitamin B_9_) is a water-soluble vitamin that plays an important role in growth, health, and nutrient metabolism. Folic acid is necessary for one-carbon transfer metabolic reactions, including nucleic acid synthesis, amino acid metabolism, and remethylation (conversion of homocysteine to methionine; Hayashi et al. 2007). Therefore, folic acid plays an important role in protein deposition and tissue synthesis. However, before folic acid can serve as a coenzyme and be used for methionine metabolism and protein synthesis, it first needs to be reduced and biochemically activated. This occurs in the liver and requires dihydrofolate reductase (DHFR) and other B vitamins (Raghubeer and Matsha 2021).

Additionally, folic acid is necessary for proper brain function. It is also needed for the production of DNA and RNA. This is especially important during periods of rapid cell and tissue growth, such as pregnancy, infancy, and adolescence in humans (Jiang et al. 2024). In swine, folic acid is commonly supplemented in sow diets to improve sow reproductive performance by positively impacting embryo development and increasing the number of pigs born alive (Matte et al. 1984; Thaler et al. 1989; Guay et al. 2002). An industry survey conducted by Faccin et al. (2023) found a range of 0.44 to 20.6 mg/kg of added folic acid in diets from weaning to 7 kg. While the NRC (2012) suggests nursery pigs from 5 to 25 kg body weight (BW) require 0.30 mg/kg of dietary folic acid, recent research with weanling pigs supplemented up to 18 mg/kg folic acid in diets without antibiotics and pharmacological levels of Zn observed a shift in short-chain fatty acid (SCFA) metabolism (Wang et al. 2021a). The SCFA shifted towards improved energy efficiency by increasing the isobutyrate concentration which can be used as an energy source for colonocytes (Jaskiewicz et al. 1996). The shift in SCFA metabolism and increased cecum crypt depth might have been responsible for increased average daily gain (ADG) found with increased dietary folic acid (Wang et al. 2020, 2021a, b).

Zinc is known to have antibacterial properties (Sirelkhatim et al. 2015). Pharmacological levels of Zn from zinc oxide (ZnO) in nursery pig diets have been observed to reduce the incidence of diarrhea and improve growth performance (Carlson et al. 1999; Hill et al. 2001; Laskoski et al. 2021). Because of the increased ADG observed by Wang et al. (2020, 2021a,b) with supra-nutritional additions of folic acid in nursery pig diets without pharmacological levels of Zn and antibiotics, the objective for Exp. 1 was to determine the effects of including folic acid with or without pharmacological levels of Zn on nursery pig growth performance and fecal dry matter (DM). The hypothesis was that growth performance would increase with the addition of folic acid but may be dependent on Zn level of the diet.

Based on the results of Exp. 1, a second experiment was conducted to further understand the effect of folic acid on growth performance and its role in protein synthesis, specifically the remethylation of homocysteine to methionine. The objective was to validate results of Exp. 1 and determine if serum homocysteine played a role in this response. Based on the results of Exp. 1, the hypothesis was that excess folic acid and increased serum homocysteine concentrations may result in poorer growth performance.

## Materials and methods

### General

The Kansas State University Institutional Animal Care and Use Committee approved the protocol used in this experiment. Experiment 1 was conducted at the Kansas State University Swine Teaching and Research Center in Manhattan, KS. Each pen (1.52 × 1.52 m) had metal tri-bar flooring, one 4-hole self-feeder, and a nipple waterer to provide ad libitum access to feed and water. Experiment 2 was conducted at the Kansas State University Segregated Early Weaning Research Facility in Manhattan, KS. Each pen (1.22 × 1.22 m) had metal tri-bar floors, one 4-hole self-feeder, and nipple waterer for ad libitum access to feed and water. The facilities are completely enclosed, environmentally controlled, and mechanically ventilated.

### Experiment 1

A total of 360 barrows (DNA 600 × 241; initially 5.5 ± 0.03 kg) were used in a 38-d growth study to evaluate the effects of including folic acid with or without pharmacological levels of Zn (from ZnO) on growth performance and fecal DM in nursery pigs. Diets did not contain any feed-grade medications. Pigs were weaned at approximately 19 ± 1.4 d of age and placed in pens with 5 pigs per pen. Each pen contained one pig from each 20^th^ percentile of the population to ensure the distribution of weight in each pen was reflective of the entire population. Following allotment to pen, pens were randomly allotted to one of six dietary treatments in a completely randomized design. A total of 72 pens were used with 12 replications per treatment. Dietary treatments were arranged in a 3 × 2 factorial with main effects of folic acid (0, 20, or 40 mg/kg; Rovimix Folic Acid, dsm-firmenich, Plainsboro, NJ; Table 1) and Zn provided by ZnO (3000 mg/kg of Zn from d 0 to 9 and 2000 mg/kg from d 9 to 24, or no additional Zn above that provided in the trace mineral premix). Experimental diets were corn-soybean meal-based with added specialty lactose and protein sources and fed in 2 phases in meal form (phase 1 was fed from d 0 to 9 and phase 2 was fed from d 9 to 24). A common phase 3 diet was fed to all pigs in meal form from d 24 to 38. All diets contained 110 mg/kg of Zn provided by the trace mineral premix, but the premixes did not contain folic acid. At the time of feed manufacturing, a single base diet was manufactured at Hubbard Feeds in Beloit, KS, packaged in 22.7 kg bags and then transported to O.H. Kruse Feed Technology Innovation Center at Kansas State University, Manhattan, KS, where treatment-specific ingredients were mixed with the base diet to form experimental treatments. Pigs were weighed on d 0, 9, 24, and 38 to determine average daily gain (ADG), average daily feed intake (ADFI), and gain: feed (G: F).

**Table 1:** Diet composition (as-fed basis), exp. 1^a^.

Ingredient, %	Phase 1^b^	Phase 2^c^	Phase 3^d^
**Corn**	44.97	49.90	64.24
**Soybean meal (47.7% CP)**	17.34	23.55	31.78
**Bovine blood plasma**	2.00	…	…
**DDGS**	5.00	7.50	…
**Fish meal**	2.50	…	…
**Whey powder**	10.00	…	…
**Whey permeate**	10.00	10.00	…
**Microbial-enhanced soy protein^e^**	4.00	4.00	…
**Choice white grease**	1.00	1.00	…
**Calcium carbonate**	0.45	0.75	0.90
**Monocalcium *P* (21% P)**	0.80	1.00	1.00
**Salt**	0.30	0.50	0.60
**L-Lys-HCl**	0.40	0.55	0.50
**DL-Met**	0.18	0.22	0.21
**L-Thr**	0.17	0.22	0.21
**L-Trp**	0.02	0.04	0.04
**L-Val**	0.08	0.12	0.13
**Vitamin and trace mineral premixes^f^**	0.40	0.40	0.40
**Zinc oxide^g^**	+/−	+/−	…
**Folic acid^h^**	+/−	+/−	…
**Total**	100	100	100
**Calculated analysis**			
** SID AA, %**			
** Lys**	1.35	1.35	1.35
** Ile: Lys**	57	57	56
** Leu: Lys**	120	118	115
** Met: Lys**	35	37	36
** Met and cys: Lys**	58	58	58
** Thr: Lys**	64	63	63
** Trp: Lys**	19.2	19.3	19.4
** Val: Lys**	70	70	70
** Total Lys, %**	1.51	1.51	1.50
** NE, kcal/kg**	2553	2500	2418
** SID Lys: NE, g/Mcal**	5.83	6.15	5.68
** CP, %**	21.5	21.5	21.3
** Ca, %**	0.66	0.66	0.71
** STTD P, %**	0.58	0.51	0.47

a Dietary treatments were arranged in a 3 × 2 factorial with main effects of folic acid and pharmacological levels of Zn.

b Phase 1 diets were fed from d 0 to 9 (6 to 7 kg).

c Phase 2 diets were fed from d 9 to 24 (7 to 12 kg).

d A common phase 3 diet was fed to all pigs from d 24 to 38 (12 to 20 kg).

e ME-PRO, Prairie AquaTech, Brookings SD.

f Provided per kg of diet: 4134 IU vitamin A; 1653 IU vitamin D; 44 IU vitamin E; 3 mg vitamin K; 0.03 mg vitamin B_12_; 50 mg niacin; 28 mg pantothenic acid; 8 mg riboflavin; 110 mg Zn from zinc sulfate; 110 mg Fe from iron sulfate; 33 mg Mn from manganese oxide; 17 mg Cu from copper sulfate; 0.30 mg I from calcium iodate; 0.30 mg Se from sodium selenite. Ronozyme HiPhos (dsm-firmenich, Plainsboro, NJ) included at 1250 FYT/kg provided an estimated release of 0.13% STTD P.

g Zinc oxide was included in the diet at the expense of corn to provide 3000 mg/kg of Zn in phase 1 and 2000 mg/kg in phase 2 in the treatment groups containing pharmacological levels of Zn from ZnO.

h Rovimix Folic Acid (dsm-firmenich, Plainsboro, NJ) included at the expense of corn at 0.0025% or 0.005% of the diet for the respective treatments to provide 0, 20, or 40 mg/kg folic acid.

CP = Crude protein.

NE, net energy.

SID, standardized ileal digestibility.

STTD, standardized total tract digestibility.

### Fecal characteristics

On d 9 and 24 of the experiment, fecal samples were collected from 3 pigs per pen. Samples were collected into sealable plastic bags for each individual pig. Fecal DM was analyzed independently on all 3 samples per pen for both collection days. Fecal samples were stored in a freezer (−20°C) until analysis. Fecal samples were dried at 55°C for 48 h in a forced air oven, and the ratio of dried to wet fecal weight determined the percentage fecal DM. All 3 sample results were combined to calculate a pen value for statistical analysis.

The SCFA profiles were measured using a gas chromatographic method as described by Zhou et al. (2017) with minor modifications. Briefly, 1 g of sample was suspended in 3 mL of distilled water and settled for 60 min. Afterward, the sample was centrifuged (15,000 × *g*) at 4°C for 10 min. The 1 mL supernatant was transferred and mixed with 0.25 mL metaphosphoric acid. The sample rested overnight and then centrifuged (3000 × *g*) for 10 min. The supernatant (0.4 mL) was transferred to gas chromatography vials and analyzed using an Agilent 7890 Gas Chromatography (Agilent Technologies, Santa Clara, CA).

### Experiment 2

A total of 350 barrows (DNA 200 × 400; initially 6.0 ± 0.06 kg) were used in a 38-d growth trial. Pigs were weaned at approximately 21 ± 1.4 d of age and randomly allotted to 1 of 5 dietary treatments in a completely randomized design. A total of 70 pens were used with 5 pigs per pen placed in pens in a similar manner to Exp. 1 with 14 replications per treatment. Dietary treatments were corn-soybean meal-based with added specialty lactose and protein sources, and consisted of increasing folic acid: 0, 5, 10, 20, or 40 mg/kg (Rovimix Folic Acid, dsm-firmenich, Plainsboro, NJ; Table 2). The vitamin premix used in these diets did not contain folic acid. Treatment diets were fed in three phases from d 0 to 10 (phase 1), d 10 to 23 (phase 2), and d 23 to 38 (phase 3) after weaning. Treatment diets were manufactured at the Kansas State University O.H. Kruse Feed Technology Innovation Center in Manhattan, KS. Phase 1 diets were fed in pelleted form. Phase 2 and 3 diets were fed in meal form. Complete diet samples were taken using a grain probe during bagging from every fourth bag and pooled into one homogenized sample per dietary treatment, then stored at −20°C. Pigs were weighed on d 0, 10, 23, and 38 to determine average daily gain (ADG), average daily feed intake (ADFI), and gain: feed (G: F).

**Table 2:** Diet composition (as-fed basis), exp. 2^a^.

Ingredient, %	Phase 1^b^	Phase 2^c^	Phase 3^d^
**Corn**	43.08	53.55	65.73
**Soybean meal (47.7% CP)**	17.25	23.36	30.52
**Bovine blood plasma**	2.00	…	…
**DDGS**	5.00	7.50	…
**Fish meal**	2.50	…	…
**Whey powder**	10.00	…	…
**Whey permeate**	10.00	5.50	…
**Microbial-enhanced soy protein^e^**	5.00	5.00	…
**Soybean oil**	2.00	1.00	…
**Calcium carbonate**	0.40	0.70	0.75
**Monocalcium *P* (21% P)**	0.75	1.00	0.90
**Salt**	0.30	0.50	0.60
**L-Lys-HCl**	0.40	0.55	0.48
**DL-Met**	0.18	0.21	0.19
**L-Thr**	0.17	0.23	0.21
**L-Trp**	0.03	0.04	0.04
**L-Val**	0.08	0.12	0.11
**Vitamin and trace mineral premixes^f^**	0.40	0.40	0.40
**Zinc oxide**	0.40	0.26	…
**Folic acid^g^**	+/−	+/−	+/−
**Total**	100	100	100
**Calculated analysis**			
** SID AA, %**			
** Lys**	1.35	1.35	1.30
** Ile: Lys**	56	56	56
** Leu: Lys**	118	119	117
** Met: Lys**	35	37	36
** Met and Cys: Lys**	57	57	57
** Thr: Lys**	64	64	64
** Trp: Lys**	19.3	19.0	19.2
** Val: Lys**	69	70	69
** Total Lys, %**	1.51	1.51	1.44
** NE, kcal/kg**	2595	2485	2429
** SID Lys: NE, g/Mcal**	5.20	5.43	5.35
** CP, %**	21.2	21.5	20.8
** Ca, %**	0.66	0.66	0.65
** STTD P, %**	0.58	0.51	0.46

a Dietary treatments consisted of increasing added folic acid.

b Phase 1 diets were fed from d 0 to 10 (6 to 7 kg).

c Phase 2 diets were fed from d 10 to 23 (7 to 13 kg).

d Phase 3 diets were fed from d 23 to 38 (13 to 22 kg).

e HP 300, Hamlet Protein, Findlay, OH.

f Provided per kg of diet: 4134 IU vitamin A; 1653 IU vitamin D; 44 IU vitamin E; 3 mg vitamin K; 0.03 mg vitamin B_12_; 50 mg niacin; 28 mg pantothenic acid; 8 mg riboflavin; 110 mg Zn from zinc sulfate; 110 mg Fe from iron sulfate; 33 mg Mn from manganese oxide; 17 mg Cu from copper sulfate; 0.30 mg I from calcium iodate; 0.30 mg Se from sodium selenite. Ronozyme HiPhos (dsm-firmenich, Plainsboro, NJ) included at 1250 FYT/kg provided an estimated release of 0.13% STTD P.

g Rovimix Folic Acid (dsm-firmenich, Plainsboro, NJ) included at the expense of corn at 0.0006%, 0.0013%, 0.0025%, or 0.005% of the diet for the respective treatments to provide 5, 10, 20, or 40 mg/kg folic acid.

CP = Crude protein.

NE, net energy.

SID, standardized ileal digestibility.

STTD, standardized total tract digestibility.

### Blood characteristics

On d 10 and 23, blood samples were collected from one average weight barrow from each pen (14 per treatment) for analysis of serum homocysteine concentration. A blood sample was taken from the jugular vein using a red-top, no anti-coagulant blood tube (Mansfield, MA). Ten mL of blood was centrifuged (3000 × *g*, 30 min) within 2 h after collection, and serum was stored at −20°C until analysis. Serum homocysteine concentrations were determined using ELISA kits per the instructions of the manufacturer (Aspira Chemical, Oakland, CA).

### Statistical analysis

Growth performance data were analyzed as a completely randomized design using the GLIMMIX procedure of SAS (SAS Institute Inc., Cary, NC). Pen was considered the experimental unit and treatment was used as the fixed effect when evaluating growth performance. Linear and quadratic contrasts were evaluated within increasing folic acid level. Main effects of Zn and the interaction between Zn and increasing folic acid were evaluated in Exp 1. Fecal DM and SCFA (Exp. 1) were analyzed as repeated measures representing multiple observations on each pen over time, and pen nested within treatment was included in the model as a random intercept to account for subsampling attributed to the multiple observations for each experimental unit on each day. Treatment, day, and the associated interactions were considered fixed effects. In Exp. 2, homocysteine concentrations were analyzed as repeated measures with pen included in the model as a random intercept to account for each sample being analyzed in duplicate, and microplate was used as a random effect. Treatment, day, and the associated interaction were considered fixed effects. Contrast coefficients for Exp. 2 were generated using PROC IML to account for uneven treatment spacing. All mortality data were analyzed using a binomial distribution using a logit link function. Calculation of ADG and ADFI was based on pig days, and pigs resulting in mortality within the experiment had the number of days they were present within pen included in this calculation. Results were considered significant with *P ≤* 0.05 and were considered a tendency with *P ≤* 0.10.

## Results

### Experiment 1

No folic acid × pharmacological levels of Zn interactions were observed throughout the study (Table 3). For phase 1 (d 0 to 9), BW and G: F decreased then returned to control values (quadratic, *P ≤* 0.072) as folic acid supplementation levels increased. For phase 2 (d 9 to 24), experimental period (d 0 to 24), common period (d 24 to 38) and overall (d 0 to 38), BW, ADG, and ADFI decreased then returned to control values (quadratic, *P ≤* 0.049) as folic acid supplementation levels increased. No differences were observed in G: F or mortality based on folic acid supplementation. Numerical differences in mortality were observed with pigs fed no added folic acid having 0% mortality compared to pigs fed 20 or 40 mg/kg having 10% or 7.5% mortality, respectively.

**Table 3:** The effects of added folic acid and pharmacological levels of Zn on nursery pig growth performance, exp. 1^a^.

	Pharmacological levels of Zn^b^							*P—*value		
	No			Yes					Folic acid	
Folic acid, mg/kg^c^	0	20	40	0	20	40	SEM	Zn	Linear	Quadratic
**BW, kg**										
** d 0**	5.5	5.5	5.5	5.5	5.5	5.5	0.03	0.771	0.878	0.837
** d 9**	6.9	6.8	7.0	7.2	7.0	7.1	0.09	0.011	0.888	0.072
** d 24**	12.0	11.4	12.0	12.3	12.0	12.4	0.24	0.030	0.825	0.019
** d 38**	20.2	18.7	20.2	20.1	19.4	20.0	0.41	0.812	0.878	0.003
**d 0 to 9 (phase 1)**										
** ADG, g**	159	147	166	187	172	183	10.2	0.007	0.889	0.111
** ADFI, g**	177	172	186	207	198	207	10.6	0.004	0.645	0.315
** G: F, g/kg**	901	849	897	906	869	888	24.3	0.796	0.662	0.069
**d 9 to 24 (phase 2)**										
** ADG, g**	337	303	329	344	331	349	13.1	0.086	0.902	0.049
** ADFI, g**	459	414	447	467	444	477	15.0	0.067	0.975	0.012
** G: F, g/kg**	734	734	734	737	743	732	13.0	0.755	0.844	0.698
**d 0 to 24 (experimental period)**										
** ADG, g**	270	242	267	285	269	284	9.8	0.016	0.808	0.015
** ADFI, g**	353	318	348	369	348	371	11.8	0.019	0.896	0.009
** G: F, g/kg**	766	762	768	772	773	766	10.4	0.568	0.803	0.942
**d 24 to 38 (common period)**										
** ADG, g**	590	521	588	555	529	540	18.1	0.096	0.649	0.008
** ADFI, g**	835	748	830	808	781	793	22.1	0.573	0.647	0.008
** G: F, g/kg**	705	698	708	686	677	682	12.0	0.028	0.953	0.452
**d 0 to 38**										
** ADG, g**	388	342	382	385	360	375	11.2	0.761	0.497	0.002
** ADFI, g**	531	471	521	531	499	522	15.0	0.439	0.522	0.002
** G: F, g/kg**	732	726	733	724	721	721	7.0	0.154	0.867	0.522
**Fecal DM, %^d^**	19.7	20.5	21.3	21.9	22.0	21.0	0.80	0.080	0.643	0.710

a A total of 360 pigs (initially 5.5 ± 0.03 kg) were used with 5 pigs per pen and 12 replications per treatment. Dietary treatments were arranged in a 3 × 2 factorial with main effects of folic acid and pharmacological Zn. No folic acid × Zn interactions were observed (*P >* 0.10).

b Zinc oxide was included in the diet to provide 3000 mg/kg of Zn from d 0 to 9 and 2000 mg/kg from d 9 to 24.

c Rovimix Folic Acid (dsm-firmenich, Plainsboro, NJ).

d Fecal samples were collected individually from 3 pigs per pen (216 pigs total) on d 9 and 24. No folic acid × Zn × day, folic acid × day, or Zn × day were observed (*P >* 0.10). Fecal DM was greater (*P = *0.007) on d 24 compared to d 9.

ADFI, average daily feed intake.

ADG, average daily gain.

BW, body weight.

DM, dry matter.

G: F, gain-to-feed ratio.

Pigs fed diets with pharmacological levels of Zn had increased (*P ≤* 0.011) d 9 BW, ADG, and ADFI compared with pigs fed 110 mg/kg added Zn (d 0 to 9). For phase 2 (d 9 to 24), pigs fed diets with pharmacological levels of Zn had increased (*P ≤* 0.086) d 24 BW, ADG, and ADFI compared with pigs fed 110 mg/kg added Zn. For the experimental period (d 0 to 24), pigs fed diets with pharmacological levels of Zn had increased (*P ≤* 0.019) ADG, and ADFI compared with pigs fed 110 mg/kg added Zn. During the common period (d 24 to 38), pigs previously fed pharmacological levels of Zn tended to have decreased ADG (*P = *0.096) and had poorer G: F (*P = *0.028) compared to pigs not previously fed pharmacological levels of Zn. Overall (d 0 to 38), no differences were observed when pigs were fed diets with or without pharmacological levels of Zn from d 0 to 24.

For fecal DM, no treatment × day or folic acid × Zn interactions were observed. Fecal DM was greater (*P = *0.007) on d 24 compared to d 9. There was a tendency for a main effect of pharmacological levels of Zn was observed where pigs fed diets with added Zn had increased (*P = *0.080) fecal DM averaged across d 9 and 24.

A total of 432 samples (72 samples per treatment) were analyzed for SCFA concentrations (Table 4). No folic acid × Zn × day, folic acid × day, or folic acid × Zn interactions were observed. A Zn × day interaction (*P = *0.035) was observed for isovalerate concentration from a total of 63 samples with detectable levels across all treatments. On d 9, isovalerate concentrations were greater in fecal samples of pigs fed 110 mg/kg added Zn compared to fecal samples from pigs fed pharmacological Zn (0.84 vs. 0.33 µmol/g, respectively; *P = *0.008). On d 24, there was no difference in isovalerate concentrations between Zn treatments (0.44 vs. 0.50 234 µmol/g, respectively). A tendency for a Zn × day interaction (*P = *0.087) was also observed for isobutyrate concentration from a total of 6 samples with detectable levels across all treatments. On d 9, isobutyrate concentration was greater in fecal samples from pigs fed 110 mg/kg added Zn compared to fecal samples from pigs fed pharmacological Zn (0.08 vs. 0.0003 µmol/g, respectively). On d 24, there was no difference in isobutyrate concentrations between Zn treatments (0.02 vs. 0.04 µmol/g, respectively). Additionally, increasing folic acid increased (linear, *P = *0.030) isobutyrate concentrations. Pigs fed pharmacological Zn had increased (*P = *0.044) valerate concentrations compared to those fed 110 mg/kg added Zn from a total of 84 samples with detectable levels across all treatments. Acetate, valerate, and total SCFA concentrations were greater (*P ≤* 0.053) on d 24 compared with values on d 9.

**Table 4:** The effects of folic acid and pharmacological levels of Zn on nursery pig fecal characteristics, exp. 1^a^.

Pharmacological levels of Zn^b^											
	No			Yes				Day			
Folic acid, mg/kg^c^	0	20	40	0	20	40	SEM	9	24	SEM	*P* =
**Fecal DM, %^d^**	19.7	20.5	21.3	21.9	22	21	0.8	20.3	21.8	0.42	0.007
**SCFA^e^**											
** Acetate, μmol/g**	55.2	51.3	51	52	53.6	51.1	3.76	51	54.7	1.91	0.042
** N^f^**	67	63	64	63	66	61	…	190	194	…	…
** Butyrate, μmol/g**	7.1	5.9	6.9	6.8	7	6	1.01	6.3	6.9	0.508	0.28
** N^f^**	48	41	47	44	46	39	…	122	143	…	…
** Propionate, μmol/g**	19.8	18.4	18.8	18.2	19.5	18	1.25	18.3	19.3	0.64	0.231
** N^f^**	65	61	63	63	66	59	…	183	194	…	…
** Isovalerate, μmol/g^g^**	0.54	0.77	0.62	0.27	0.38	0.59	0.172	0.58	0.47	0.096	0.388
** N^f^**	10	13	11	6	10	13	…	33	30	…	…
** Valerate, μmol/g^h^**	0.75	1.04	1.18	0.43	0.38	0.75	0.283	0.55	0.97	0.133	0.003
** N^f^**	15	19	19	8	9	14	…	26	58	…	…
** Isobutyrate, μmol/g^i^**	0	0.03	0.12	0	0.03	0.03	0.036	0.04	0.03	0.021	0.697
** N^f^**	0	1	3	0	1	1	…	3	3	…	…
** Total SCFA, μmol/g**	83.6	77.4	78.7	77.8	80.8	76.5	5.36	75.9	82.4	2.74	0.053

a A total of 360 pigs (initially 5.5 ± 0.03 kg) were used with 5 pigs per pen and 12 replications per treatment. Dietary treatments were arranged in a 3 × 2 factorial with main effects of folic acid and zinc oxide. Fecal samples were collected individually from 3 pigs per pen (216 pigs total) on d 9 and 24. No folic acid × Zn × day, folic acid × day, Zn × day, or folic acid × Zn interactions were observed (*P >* 0.10) unless otherwise noted.

b Zinc oxide was included in the diet to provide 3000 mg/kg of Zn from d 0 to 9 and 2000 mg/kg from d 9 to 24.

c Rovimix Folic Acid (dsm-firmenich, Plainsboro, NJ).

d A marginally significant main effect of pharmacological levels of Zn was observed where pigs fed diets with added Zn had increased (*P = *0.080) fecal DM.

e SCFA = short chain fatty acids. Concentrations were measured using a gas chromatographic method as described by Zhou et al. (2017) with minor modifications.

f A total of 72 samples per treatment were analyzed. Values represent the number of samples with detectable SCFA. Samples without detectable SCFA were considered as 0.00 concentration for analysis. There were no samples with detectable levels of isocaproate, caproate, or heptanoate.

g Zn × day, *P = *0.035. On d 9, isovalerate was greater in fecal samples from pigs not fed pharmacological Zn compared to fecal samples from pigs fed pharmacological Zn (0.84 vs. 0.33 μmol/g, respectively; *P = *0.008). On d 24, there was no difference in isovalerate between Zn treatments (0.44 vs. 0.50 μmol/g, respectively; *P = *0.781).

h Main effect of ZnO, *P = *0.044.

i Zn × day, *P = *0.087. On d 9, isobutyrate was greater in fecal samples from pigs not fed pharmacological Zn compared to fecal samples from pigs fed pharmacological Zn (0.08 vs. 0.0003 μmol/g, respectively; *P = *0.052). On d 24, there was no difference in isobutyrate between Zn treatments (0.02 vs. 0.04 μmol/g, respectively; *P = *0.577). Linear effect of folic acid, *P = *0.030.

### Experiment 2

For phase 1 (d 0 to 10), there were no differences in d 10 BW, ADG, or ADFI with increasing folic acid supplementation levels; however, increasing folic acid supplementation levels decreased (linear, *P = *0.032; Table 5) G: F. For phase 2 (d 10 to 23), d 23 BW, ADG, and ADFI decreased then increased (quadratic, *P ≤* 0.079) as folic acid supplementation levels increased, with pigs fed 20 mg/kg having the poorest performance. No treatment differences were observed for G: F. For phase 3 (d 23 to 38) and overall (d 0 to 38), BW, ADG, ADFI, and G: F decreased then increased (quadratic, *P ≤* 0.047) as folic acid supplementation levels increased, but similar to d 10 to 23, pigs fed 20 mg/kg of folic acid had the poorest performance.

**Table 5:** Effects of added folic acid on nursery pig growth performance, exp. 2^a^.

	Folic acid, mg/kg^b^						*P—*value	
Item	0	5	10	20	40	SEM	Linear	Quadratic
**BW, kg**								
** d 0**	6.0	6.0	6.0	6.0	6.0	0.06	0.759	0.482
** d 10**	7.4	7.4	7.3	7.1	7.2	0.20	0.115	0.255
** d 23**	12.8	12.5	12.7	12.1	12.4	0.21	0.068	0.069
** d 38**	22.5	21.8	21.9	20.9	21.6	0.54	0.023	0.002
**d 0 to 10 (phase 1)**								
** ADG, g**	138	139	139	115	125	15.4	0.127	0.315
** ADFI, g**	142	142	142	131	139	9.4	0.494	0.375
** G: F, g/kg**	959	974	968	868	886	57.1	0.032	0.458
**d 10 to 23 (phase 2)**								
** ADG, g**	421	392	406	384	397	10.2	0.203	0.079
** ADFI, g**	547	515	535	494	507	11.0	0.015	0.055
** G: F, g/kg**	770	761	757	779	783	13.0	0.148	0.780
**d 23 to 38 (phase 3)**								
** ADG, g**	642	624	616	587	618	24.9	0.059	0.001
** ADFI, g**	911	892	890	851	879	38.0	0.025	0.005
** G: F, g/kg**	706	699	693	689	703	8.9	0.948	0.049
**d 0 to 38**								
** ADG, g**	434	417	417	393	413	12.7	0.031	0.002
** ADFI, g**	584	566	570	539	557	17.6	0.014	0.008
** G: F, g/kg**	742	737	732	729	741	5.6	0.966	0.047

a A total of 350 barrows (initially 6.0 ± 0.06 kg) were used with 5 pigs per pen and 14 replications per treatment.

b Rovimix Folic Acid (dsm-firmenich, Plainsboro, NJ).

ADFI, average daily feed intake.

ADG, average daily gain.

BW, body weight.

DM, dry matter.

G: F, gain-to-feed ratio.

A marginally significant linear treatment × day interaction was observed (*P = *0.069) for serum homocysteine concentrations (Table 6). On d 10, an increase (linear, *P = *0.037) in homocysteine concentration was observed as folic acid supplementation levels increased. However, no differences were observed among treatments on d 23. Pigs had greater (*P <* 0.001) serum homocysteine concentrations on d 10 compared to d 23.

**Table 6:** Effects of folic acid on nursery pig serum homocysteine concentrations, exp. 2^a^.

	Folic acid, mg/kg^b^						*P—*value	
	0	5	10	20	40	SEM	Linear treatment × day^c^	Folic acid, linear
**Homocysteine, umol/L^c^**								
** d 10**	19.2	20.9	22.2	26.9	29.8	4.70	0.069	0.037
** d 23**	14.5	17.9	15.2	24.8	17.7	…	…	0.450

a A total of 350 barrows (initially 6.0 ± 0.06 kg) were used with 5 pigs per pen and 14 replications per treatment. Blood samples were collected from one average weight barrow from each pen on d 10 and 23.

b Rovimix Folic Acid (dsm-firmenich, Plainsboro, NJ).

c Quadratic folic acid × day interaction also tested, *P = *0.193. Main effect of day, *P <* 0.001.

## Discussion

Folic acid is necessary for one-carbon transfer metabolic reactions, including nucleic acid synthesis, amino acid metabolism, and remethylation (Hayashi et al. 2007). Folic acid must be provided in the diet either from the ingredients or added in a vitamin premix. For folic acid to be used in one-carbon transfer reactions, including methionine metabolism, and act as a coenzyme, it needs to be reduced and biochemically activated. This involves a 2-step reduction with the conversion of folic acid to dihydrofolate (DHF) which is then converted to tetrahydrofolate (THF). Both conversions are facilitated by the enzyme DHFR and occur in the liver (Scaglione and Panzavolta 2014; Raghubeer and Matsha 2021). Folic acid must then be biochemically activated involving THF to be converted to 5,10-methyleneTHF and then converted to 5-methylTHF via 5,10-methylenetetrahydrofolate reductase (MTHFR; Raghubeer and Matsha 2021). Five-methylTHF is used as a methyl donor in pyrimidine and purine synthesis and can donate a methyl group to convert homocysteine to methionine. The conversion reaction of homocysteine to methionine is known as remethylation and is catalyzed by methionine synthase with vitamin B_12_ as a cofactor (Raghubeer and Matsha 2021). The reduction and activation steps for folic acid metabolism are essential for its utilization in methionine metabolism and protein synthesis.

In the current experiment, supra-nutritional additions of folic acid up to 40 mg/kg were evaluated. The NRC (2012) requirement estimate for folic acid in 5 to 25 kg pigs is 0.30 mg/kg. However, the requirement estimate was established based on research in the 1980’s and may not be representative of today’s genetics (Easter et al. 1983; Lindemann and Kornegay 1986). Recently, an industry survey by Faccin et al. (2023) observed a range of 0.44 to 20.6 mg/kg of added folic acid in diets from weaning to 7 kg. Generally, vitamin toxicity is low for water-soluble B-vitamins compared with fat-soluble vitamins (Rafeeq et al. 2020). Wang et al. (2020, 2021a, b). Weanling pigs fed up to 18 mg/kg of added folic acid observed a linear increase in ADG associated with increased cecum crypt depth, increased organ weight of liver, spleen, kidneys, and altered SCFA concentrations in the colon observed via increased isobutyrate concentration. The shift in SCFA metabolism results in increased energy efficiency because isobutyrate can be used as an energy source for colonocytes (Jaskiewicz et al. 1996).

An increase in SCFA concentrations can promote the intestinal health of weaned pigs and help mitigate the negative effects of weaning on the intestinal tract. The SCFAs play a crucial role in maintaining the morphology and function of intestinal epithelial cells, gut microbiota balance, and intestinal immune response (Liu et al. 2018). The SCFAs provide 60% to 70% of the energy for the colonic epithelium, with butyrate being the most energy-efficient SCFA, followed by propionate and acetate (Roediger 1982). Additionally, SCFAs can inhibit the colonization and growth of *Salmonella* and *Escherichia coli* by lowering the pH of the large intestine (Shibata et al. 2017). Wang et al. (2021a) observed an increase in isobutyrate, isovalerate, and valerate concentrations in the colon when folic acid levels increased from 0 to 18 mg/kg. We observed a similar increase in isobutyrate concentration. The shift in SCFA metabolism may result in increased energy efficiency because isobutyrate can be used as an energy source for colonocytes (Jaskiewicz et al. 1996). However, in the current experiment, most pigs had nondetectable levels of isobutyrate. Within the samples that had detectable levels, the concentrations were low. In the current study, the small increase in isobutyrate concentration did not translate to increased growth performance.

There is limited data evaluating supra-nutritional additions of folic acid for weanling pigs; therefore, Exp. 1 was designed to investigate folic acid additions of 0, 20, or 40 mg/kg with or without pharmacological additions of Zn. Because results of Wang et al. (2020, 2021a, b) were linear, the current experiment fed up to 40 mg/kg folic acid to determine if there would be further improvements in growth performance beyond the levels previously evaluated. Pharmacological levels of Zn are known to reduce diarrhea and increase growth performance (Carlson et al. 1999; Hill et al. 2001; Laskoski et al. 2021). The studies by Wang et al. (2020, 2021a, b) were conducted in diets without pharmacological levels of Zn. Consequently, by including added Zn in the factorial arrangement, the attempt was to determine if the response to folic acid was dependent on the Zn level in the diet.

A direct physiological interaction between dietary Zn (including pharmacological concentration of ZnO) and folic acid is biologically plausible but not clearly supported by current evidence in pigs. Classic studies in mammalian systems have shown that folate and Zn can influence each other’s intestinal transport; in vitro and in vivo work demonstrated that the presence of zinc may inhibit folic acid uptake and vice versa at the intestinal level, suggesting a mutual inhibition when Zn and folate exist at the site of intestinal transport (Ghishan et al. 1986). However, similar to the current results, human and rodent feeding trials typically report no adverse impact of folic acid on zinc status or of zinc on folate utilization when both nutrients are consumed at physiological levels. In weaned piglets, pharmacological concentrations of ZnO primarily affects intestinal microbiota composition, with positive influences on intestinal functionality (Schokker et al. 2023) but no specific evidence that ZnO directly alters folate metabolism or absorption in this context. Given that both Zn and folate contribute to gut development and nutrient metabolism independently, any interaction is more likely to be indirect and context-dependent (e.g., mediated through changes in microbial fermentation or intestinal epithelial turnover) rather than a straightforward nutrient to nutrient interaction. Consequently, while mechanistic studies raise the possibility of modulation at the intestinal level, empirical evidence for a consistent Zn × folic acid interaction affecting growth performance in swine remains limited.

Based on results of Exp. 1, graded levels of folic acid (0, 5, 10, 20, and 40 mg/kg) were fed to determine a dose-response curve. Results of both experiments reported herein observed decreased growth performance contradicting the linear increase observed by Wang et al. (2020, 2021b). We are unsure as to why the different responses were observed in the current study and results of Wang et al. (2020, 2021a, b). The experimental diets used in the current study had a similar formulation strategy to Wang et al. (2020, 2021a, b) and were based on corn and soybean meal with added lactose sources, and similar concentrations of other vitamins. The different responses observed in the present experiments compared with those reported by Wang et al. (2020, 2021a, b) may be related to the greater initial BW of pigs used in their studies relative to those in the current experiments. In addition, differences in pigs genetics among studies and the duration of feeding elevated folic acid diets may have contributed to the divergent results.

In both experiments herein, a quadratic response was observed for growth performance with a reduction observed in pigs fed between 0 and 20 mg/kg of folic acid followed by an increase from 20 to 40 mg/kg. Contrary to these results, Gao et al. (2023) observed a tendency for numerical improvement in ADG when dietary folic acid was increased from 0 to 15 mg/kg. Similarly, Yu et al. (2010) reported that ADG was maximized in weaned pigs fed diets containing folic acid between 0.5 and 5 mg/kg. Consistent with the current results, Christensen et al. (2015) demonstrated in mice that high folic acid intake impaired one-carbon metabolism and was associated with hepatocellular degeneration and disruptions in lipid and phospholipid metabolism, indicating a reduced capacity of tissues to accommodate metabolic stress under conditions of folic acid oversupply. Such metabolic disturbances could compromise cellular function and energy utilization, providing a plausible biological basis for the reduced growth performance observed at supranutritional folic acid concentrations. However, it remains unclear why the response observed in the current experiment differs from most previous studies evaluating increasing levels of dietary folic acid in pigs.

The reduction in growth performance observed between 0 and 20 mg/kg of folic acid was sustained throughout the duration of the trial. However, the slight increase in performance from 20 to 40 mg/kg of folic acid may be due to the body excreting more folic acid at 40 mg/kg compared to 20 mg/kg. Because folic acid is a water-soluble vitamin, excess is excreted in urine. Folic acid is filtered in the glomerulus and is reabsorbed in the proximal renal tubule (Bailey and Ayling 2009). When high folic acid doses are fed, more folic acid is excreted in the urine due to exceeding the capacity of the liver and kidney. However, this threshold is not clearly defined, and variation exists in literature (Bailey et al. 2015). According to the data reported herein, growth performance was reduced when fed up to 20 mg/kg of folic acid, but at 40 mg/kg growth was comparable to low folic acid concentrations. This may partially be explained by this theory of excess folic acid being excreted via urinary excretion and thus increasing the downregulation of the enzymes involved with folic acid metabolism (Yu et al. 2010).

Homocysteine is an indicator of folic acid status (Bailey et al. 2015). Serum homocysteine concentrations are controlled by two metabolic pathways: (i) remethylation of homocysteine to methionine, which requires the presence of folic acid and vitamin B_12_ as coenzymes and occurs within the cell and in all body tissues or (ii) transsulfuration of homocysteine to cysteine, which requires vitamin B_6_ (Vezzoli et al. 2020). In the current experiment, serum homocysteine concentrations increased with increasing folic acid from 0 to 40 mg/kg on d 10 post-weaning suggesting that remethylation of homocysteine to methionine did not take place as previously demonstrated by Yu et al. (2010) in nursery pigs fed diets containing folic acid ranging between 0 and 10 mg/kg. This may be explained by a downregulation of 5-methylTHF and MTHFR resulting in a decrease in methyl groups to donate. However, cysteine concentrations were not measured in the current experiment. Although a similar response was expected by day 23, the current results indicated no differences in homocysteine concentration. To the best of our knowledge, there are no available data in pigs to support this observation. However, in rats, Dev et al. (2011) demonstrated that short-term (10 d) over supplementation of folic acid significantly reduced intestinal folate uptake by decreasing the number of folate transporters. Conversely, with prolonged exposure (60 d) to elevated folic acid levels, intestinal folate transport and transporter expression remained unaltered compared to normal folic acid levels. These findings suggest that another plausible explanation for the lack of change in homocysteine concentration on d 23 could be a reduction in folate absorption at the intestinal level because of the longer supplementation period.

The enzyme DHFR is needed to reduce folic acid in the liver and convert it to a form that can be utilized for one-carbon transfer reactions. However, the liver has low activity of DHFR and therefore, limited ability to reduce folic acid (Scaglione and Panzavolta 2014). This limitation results in the inability to metabolize high doses of folic acid and leads to unmetabolized folic acid in circulation in the form of DHF. High levels of DHF can inhibit MTHFR (Smith et al. 2008). Without MTHFR and 5-methylTHF, no methyl groups are available for donation to facilitate the remethylation of homocysteine to methionine. Thus, high concentrations of homocysteine were likely observed in serum because of this limitation. High concentrations of homocysteine, or hyperhomocysteinemia, a major risk factor for the development of cardiovascular disease in humans because it can lead to endothelial cell damage and reduced flexibility of vessels (Baszczuk and Kopczyński 2014; Ganguly and Alam 2015). Hyperhomocysteinemia also alters the metabolism and mobilization of the trace minerals iron, copper, and nickel resulting in elevated iron stores in pigs (Ambrosi et al. 1999).

In Exp. 1, numerically high mortality was observed when pigs were fed 20 or 40 mg/kg of folic acid (none, 10% and 7.5% for 0, 20, and 40 mg/kg added folic acid, respectively). Folic acid is not regarded as toxic because it is a water-soluble vitamin. Further research is needed to understand if the increased mortality is related to hyperhomocysteinemia. We speculate that the increase in mortality with increasing added folic acid in Exp. 1 could be because it surpassed the liver’s capacity to reduce folic acid resulting in high amounts of unmetabolized folic acid in circulation. The activity of natural killer cells has been reported to be decreased in older women having high plasma levels of unmetabolized folic acid (Troen et al. 2006). Natural killer cells are large lymphocytes that play a role in the innate immune response mainly against viruses (Lemire et al. 2017). Natural killer cells respond via the production of pro-inflammatory cytokines. In the current study, all deceased pigs were transported to the Kansas State Veterinary Diagnostic Laboratory (Manhattan, KS) for necropsy. The necropsy reports provided evidence that *Streptococcus suis* was the leading cause of death for the pigs and is a pathogen commonly identified in this research herd. Therefore, this leads us to speculate that high folic acid supplementation decreased the activity of natural killer cells and reduced the pig’s ability to combat *Streptococcus suis* infection. However, the high mortality was not observed in Exp. 2, but it was conducted in a different facility and with a different population of pigs. Additional research is warranted to further understand the possible connection between high dietary folic acid levels and increased mortality.

Pharmacological levels of Zn from ZnO were evaluated in Exp. 1 because previous studies have observed reduced incidence of diarrhea and improved growth performance with pharmacological Zn additions in weanling pig diets (Carlson et al. 1999; Hill et al. 2001; Laskoski et al. 2021). In the current experiment, increased growth performance was observed immediately after weaning with pharmacological levels of Zn. However, the response was mostly lost during the common period when pigs were fed diets without pharmacological additions of Zn resulting in no overall benefit in final weight. Other studies have also observed a benefit to Zn during the experimental period but not sustained throughout the overall nursery period (Batson et al. 2021; Stas et al. 2024). Additionally, pharmacological levels of Zn improved fecal DM in the current experiment. This may be explained by the antibacterial properties associated with Zn and improved gut health (Sirelkhatim et al. 2015; Luise et al. 2024). Laskoski et al. (2021) and Batson et al. (2021) also observed increased fecal DM with the addition of pharmacological levels of Zn in nursery pig diets. However, the addition of pharmacological levels of Zn did not interactively affect the growth or fecal DM response to added folic acid.

In conclusion, increasing dietary folic acid from 0 to 20 mg/kg reduced growth performance in nursery pigs; however, performance returned to control levels when folic acid was increased from 20 to 40 mg/kg, and this response was independent of dietary Zn concentration. Pigs fed pharmacological levels of Zn exhibited improved growth performance during the experimental period, although this benefit was not maintained over the entire nursery period.

## List of abbreviations

ADFI: average daily feed intake
ADG: average daily gain
BW: body weight
DHF: dihydrofolate
DHFR: dihydrofolate reductase
DM: dry matter
G: F: gain-to-feed ratio
MTHFR: 5,10-methylenetetrahydrofolate reductase
NE: net energy
SCFA: short chain fatty acid
SID: standardized ileal digestibility
STTD: standardized total tract digestibility
THF: tetrahydrofolate
ZnO: zinc oxide
